# Computational Study on E-Hooks of Tubulins in the Binding Process with Kinesin

**DOI:** 10.3390/ijms23042035

**Published:** 2022-02-12

**Authors:** Yixin Xie, Lin Li

**Affiliations:** 1 Computational Science Program, The University of Texas at El Paso, El Paso, TX 79912, USA; yxie4@miners.utep.edu; 2 Department of Physics, The University of Texas at El Paso, El Paso, TX 79912, USA

**Keywords:** E-hooks, C-terminus, microtubules, alpha tubulin, beta tubulin, structure flexibility, kinesin, electrostatic force, electric field line, StructureMan

## Abstract

Cargo transport within cells is essential to healthy cells, which requires microtubules-based motors, including kinesin. The C-terminal tails (E-hooks) of alpha and beta tubulins of microtubules have been proven to play important roles in interactions between the kinesins and tubulins. Here, we implemented multi-scale computational methods in E-hook-related analyses, including flexibility investigations of E-hooks, binding force calculations at binding interfaces between kinesin and tubulins, electrostatic potential calculations on the surface of kinesin and tubulins. Our results show that E-hooks have several functions during the binding process: E-hooks utilize their own high flexibilities to increase the chances of reaching a kinesin; E-hooks help tubulins to be more attractive to kinesin. Besides, we also observed the differences between alpha and beta tubulins: beta tubulin shows a higher flexibility than alpha tubulin; beta tubulin generates stronger attractive forces (about twice the strengths) to kinesin at different distances, no matter with E-hooks in the structure or not. Those facts may indicate that compared to alpha tubulin, beta tubulin contributes more to attracting and catching a kinesin to microtubule. Overall, this work sheds the light on microtubule studies, which will also benefit the treatments of neurodegenerative diseases, cancer treatments, and preventions in the future.

## 1. Introduction

Molecular motors are essential for living organisms as they take charge of converting energy into motion or mechanical work, including cargo transport of organelles, secretory vesicles and protein complexes in an extremely efficient way, which are superior to current human-made motors [1,2]. There are three major families of cytoskeleton molecular motors: myosin, dynein and kinesin families. One of the most important functions of kinesin molecular motor proteins is carrying cargos (organelles and vesicles) from the center to the periphery of the cell, which means kinesins usually move towards the plus end of microtubules (MTs) [3]. However, some of the kinesins are found to have bidirectionality [4,5,6,7]. Normal kinee.gsin is crucial to a healthy cell, as certain types of mutations on kinesin proteins lead to nervous system disorders such as peripheral neuropathy [8].

Kinesins perform their functions by moving along on microtubules. Microtubules are cytoskeletal structures that are formed by the self-assembly of alpha-beta tubulin heterodimers (~450 amino acids each) [8,9]. They are 17 nm in interior diameter and 25 nm in the outer. They are important structural elements of cells as they serve as the supports in outlining the shape of cells [10,11]. The functions of microtubules include the axon extension in neurons and assembly of the mitotic spindle in dividing cells. Normal functioning of microtubules is essential for a healthy cell and related systems, including nervous system. The abnormalities of microtubules are associated with neurodegenerative diseases [12].

Besides, microtubules are traditional drug targets for cancer treatment. Anti-mitotic drugs are highly validated chemotherapy drugs and are widely used for cancer treatment [13,14]. Traditional microtubule targeting anti-mitotic drugs aims at depolymerizing or stabilizing microtubules to destroy the microtubule dynamic [15,16], which blocks the mitosis and then kills the overactive cancer cells.

Recent work found that another promising direction of blocking the mitosis of cancer cells is targeting kinesins. Kinesins-5 function as motors to separate microtubules and form spindles in mitosis. Interrupting the binding or motility of specific kinesins can also block the mitosis and kill the cancer cells. Due to the various types of kinesins [17], kinesin targeting drugs will be more selective and also alternative drugs to solve the drugs resistance to microtubule targeting drugs. Therefore, discovering and designing drugs targeting certain types of kinesins become a new promising direction for cancer treatment [18,19,20]. Therefore, understanding the microtubules, kinesins and their interactions is highly demanding for fundamental and disease treatment purposes.

As a part of tubulin structures, C-terminal tails of each tubulin are considered to be extremely flexible. Therefore, the structures of tails are extremely difficult to be resolved by experimental methods. The C-terminal tails of tubulins have been noticed and studied since the last century [21]. The name of E-hook was firstly defined in 2000 as it contains many glutamates (GLU, E) which are negatively charged at pH 7, and their work showed that a particular interaction related to E-hooks was essential for the one-dimension Brownian movement along MTs in the w-state [22]. In the following decades, E-hooks have been proven to play important roles in a cell, including increasing cytoplasmic dynein and kinesin processivity [23], increasing the speed by the interacts with kinesin’s head [24], providing guidance and a soft landing for the microtubule [25]. Moreover, Nuclear Magnetic Resonance (NMR) and single-molecule methods have characterized and evaluated the effects of E-hook mutations on cell function [26].

Even though E-hooks are believed to be of high importance in cytoskeletal regulations and functions, the full functions of the E-hooks are still not well understood. Here, we implemented multi-scale computational methods in E-hooks related analyses, including flexibility of E-hooks, binding force calculations at binding interfaces between kinesins and tubulins, electrostatic potential calculations on the surface of kinesins and tubulins.

Our results show that E-hooks have several functions during the binding process: E-hooks utilize their own high flexibility in a solvent to increase the chances of finding a kinesin, based on the E-hook flexibility analyses; E-hooks help tubulins to be more attractive to kinesin, based on the electrostatic calculations. Besides, we also observed the differences between alpha and beta tubulins: beta tubulin shows a higher flexibility than alpha tubulin, based on the E-hooks flexibility analyses; beta tubulin generates higher attractive forces (about twice the strength) to kinesin at different distances, no matter with E-hooks in the structure or not, based on the force calculations. Those facts may indicate that compared to alpha tubulin, beta tubulin contributes more to catching and attracting a kinesin to microtubule. Overall, this work will shed the light on microtubule studies, which will also benefit the treatments to neurodegenerative diseases and cancer treatments and preventions in the future.

## 2. Results and Discussion

First of all, the flexibilities of E-hooks in alpha and beta tubulins were calculated and compared. Secondly, we discussed the electrostatic features including electrostatic potential and electric field lines of kinesins and tubulins with/without E-hooks. Thirdly, binding forces generated in three binding interfaces (kinesin-alpha, kinesin-beta, alpha-beta) were compared and discussed.

### 2.1. Tubulins Complex Structures with/without E-Hooks

**Figure 1:**
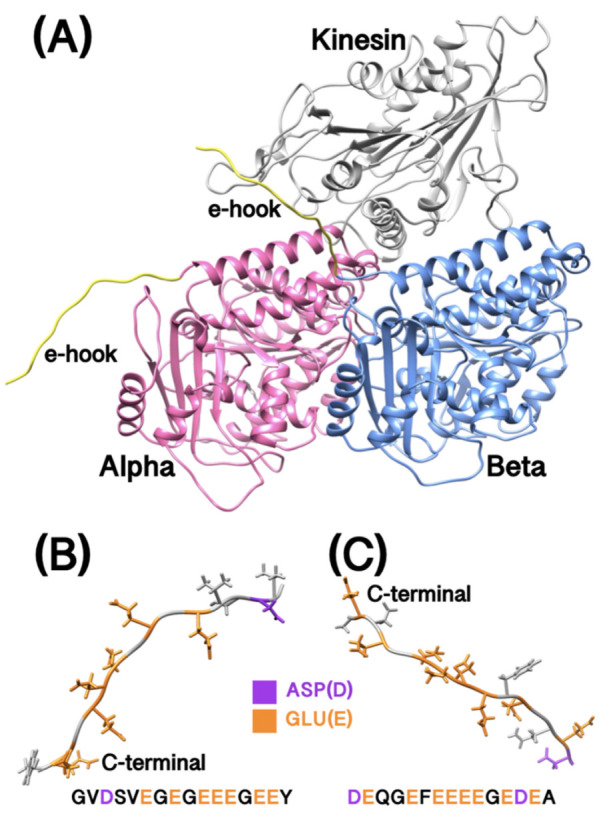
The complex structure of kinesin and tubulins with E-hooks. (**A**) The structure of kinesin and tubulins with E-hooks. Alpha tubulin is colored in pink, beta tubulin is colored in blue and kinesin is colored in grey. E-hooks in both alpha and beta tubulins are colored in yellow; (**B**) Alpha tubulin E-hook with the sequence details; (**C**) Beta tubulin E-hook with the sequence details. In B and C, GLU is colored in orange, ASP is colored in purple and residues with no charge are colored in gray.

From Figure 1B,C, the C-terminal tails (E-hooks) are rich in aspartic acid (Asp, D) and glutamic acid (Glu, E) which are negatively charged at pH 7. However, the E-hooks in alpha and beta tubulins are not the same. E-hooks in alpha tubulin have 16 AAs (GVDSVEGEGEEEGEEY) and E-hooks in beta tubulins have 15 AAs (DEQGEFEEEEGEDEA). The locations and positions of the two E-hooks are different due to the high flexibilities, which may lead to the different features between alpha and beta tubulins as discussed in the following sections.

### 2.2. Flexibility Index of E-Hooks

**Figure 2:**
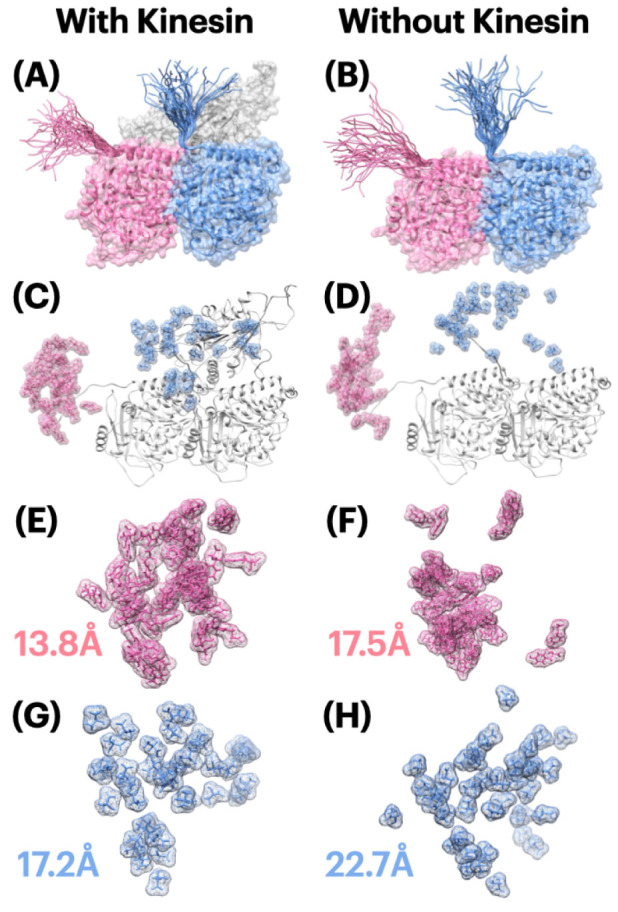
E-hooks flexibility analysis. (**A**) E-hooks moving range visualization with the presence of kinesin; (**B**) E-hooks moving range visualization without the presence of kinesin; (**C**) Moving range of the last amino acid residue of E-hooks (Y in alpha tubulin, A in beta tubulin), with the presence of kinesin; (**D**) Moving range of the last amino acid residue of E-hooks (Y in alpha tubulin, A in beta tubulin), without the presence of kinesin; (**E**) Moving range of the last amino acid residue of alpha tubulin E-hooks (Tyr, Y), with the presence of kinesin, and the flexibility index is 13.8 Å; (**F**) Moving range of the last amino acid residue of alpha tubulin E-hooks (Tyr, Y), without the presence of kinesin, and the flexibility index is 17.5 Å; (**G**) Moving range of the last amino acid residue of beta tubulin E-hooks (Ala, A), with the presence of kinesin, and the flexibility index is 17.2 Å; (**H**) Moving range of the last amino acid residue of beta tubulin E-hooks (Ala, A), without the presence of kinesin, and the flexibility index is 22.7 Å.

From Figure 2, E-hooks in both alpha and beta tubulins show a high flexibility during the simulations, with the flexibility index 13.8 Å, 17.5 Å, 17.2 Å, 22.7 Å for each scenario. By comparing the left and right columns in Figure 2, the E-hooks are more flexible in the tubulin dimer complex than in the three chains structure with a kinesin, which indicates that kinesin controls the flexibility of E-hooks. Moreover, considering one of the E-hooks’ functions is to help tubulins attract and guide a kinesin, this mechanism of regulating E-hooks’ flexibility is very efficient and energy-saving for a system, since E-hooks no longer need to stay in high flexibility after successful binding.

In terms of the differences between the E-hooks in alpha and beta tubulins, the alpha tubulin shows a lower flexibility than beta tubulin. One of the conclusions from this phenomenon is that beta tubulin contributes more to the binding process, since it has a higher flexibility of E-hooks which helps to find a kinesin in a wider range. To make this conclusion more reliable, if taking the conclusion from Section 2.5 (electrostatic binding force analyses) into consideration, beta tubulin has stronger electrostatic binding forces which shows a higher attraction to kinesin at different distances than alpha tubulin. We got a proof to support that beta tubulin plays a more important role in binding with kinesin, compared to alpha tubulin.

### 2.3. Electrostatic Potential on Surfaces

**Figure 3:**
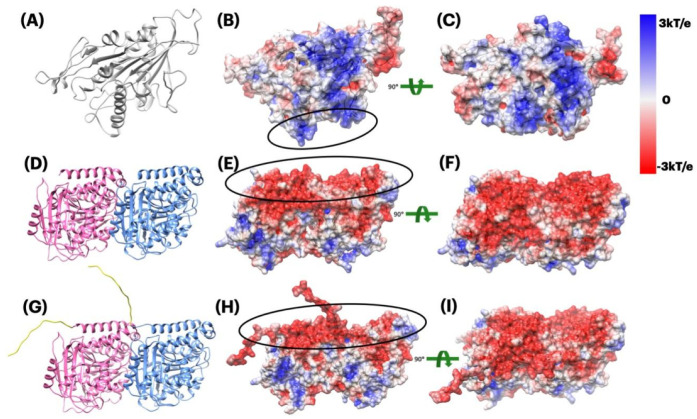
Electrostatic potential colored on the surface of kinesin and tubulins dimer. (**A**) Kinesin structure; (**B**) Electrostatic potential on the surface of kinesin (front view); (**C**) Electrostatic potential on the surface of kinesin (bottom view, interfacial side); (**D**) Tubulins dimer structure without E-hooks; (**E**) Electrostatic potential on the surface of tubulin dimer without E-hooks (front view); (**F**) Electrostatic potential on the surface of tubulin dimer without E-hooks (top view, interfacial side); (**G**) Tubulins dimer structure with E-hooks (yellow tails); (**H**) Electrostatic potential on the surface of tubulin dimer with E-hooks (front view); (**I**) Electrostatic potential on the surface of tubulin dimer with E-hooks (top view, interfacial side). Color scale: −3.0 to 3.0 kT/e (red to blue).

To study the electrostatic features, DelPhi was utilized to calculate the electrostatic potential on the surface of proteins. The electrostatic potential distribution on kinesin is shown in Figure 3B,C which was rendered by Chimera with a color scale from −3.0 to 3.0 kT/e. The charge distributions on tubulins are shown in Figure 3E,F,H,I, which were rendered by Chimera with a color scale from −3.0 to 3.0 kT/e as well. Negatively and positively charged areas are colored in red and blue, respectively.

#### 2.3.1. Electrostatic Potential of Kinesin and Tubulin Dimer

The binding interface of kinesin is overall positively charged (see the black circle in Figure 3B and blue patches in Figure 3C). The tubulin dimer, no matter with or without E-hooks is overall negatively charged (see the black circle in Figure 3E,H and red patches in Figure 3F,I).

From Figure 3, we firstly noticed the overall positively charged potential on the surface of kinesin (Figure 3B,C) and the overall positively charged potential on the surface of tubulin dimer (Figure 3E,F,H,I), which indicates that kinesin and tubulin dimer attract each other. Secondly, E-hooks (yellow tails in Figure 3G) have highly negative electrostatic potential, which makes tubulin dimer to be overall more negative, helping the tubulin to bind with kinesin more easily. Thirdly, by taking a closer look at Figure 3E,H and comparing them, E-hooks are not only negative in their local surfaces but also affect their surrounding areas to be negative, which furthermore helps the tubulin to be more attractive to kinesin at the binding interfaces.

After discussing the overall potential on tubulin dimer as a whole structure, we got interested in the individual tubulins (alpha and beta) by comparing their electrostatic potential, as follows.

#### 2.3.2. Electrostatic Potential of Alpha and Beta Tubulins

**Figure 4:**
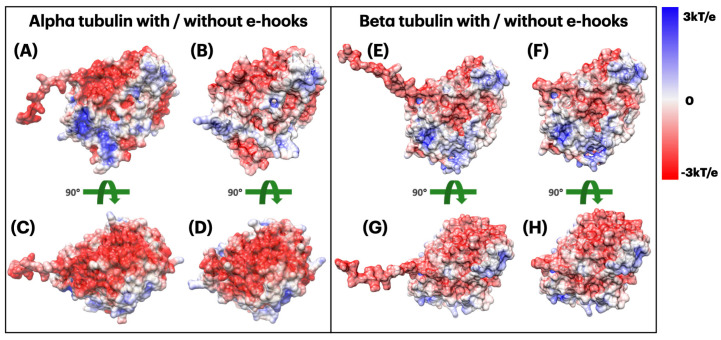
Electrostatic potential is colored on the surface of tubulins. (**A**) Alpha tubulin with E-hook (front view); (**B**) Alpha tubulin without E-hook (front view); (**C**) Alpha tubulin with E-hook (top view, interfacial side); (**D**) Alpha tubulin without E-hook (top view, interfacial side); (**E**) Beta tubulin with E-hook (front view); (**F**) Beta tubulin without E-hook (front view); (**G**) Beta tubulin with E-hook (top view, interfacial side); (**H**) Beta tubulin without E-hook (top view, interfacial side). Color scale: −3.0 to 3.0 kT/e (red to blue).

By comparing Figure 4A,B, as the E-hooks are overall negatively charged, it provides the negative potential in its surrounding area, which makes the interface more negative (Figure 4C,D). By comparing Figure 4E,F, we arrived at the similar conclusion that the E-hooks in beta tubulin also help to make the binding interface more negative. Since E-hooks help make the interface more negative and kinesin is overall positively charged, we came to the conclusion that E-hooks play important roles in supporting the binding process between kinesin and tubulins.

### 2.4. Electric Field Lines

#### 2.4.1. Electric Field Lines between Kinesin and Tubulin Dimer

**Figure 5:**
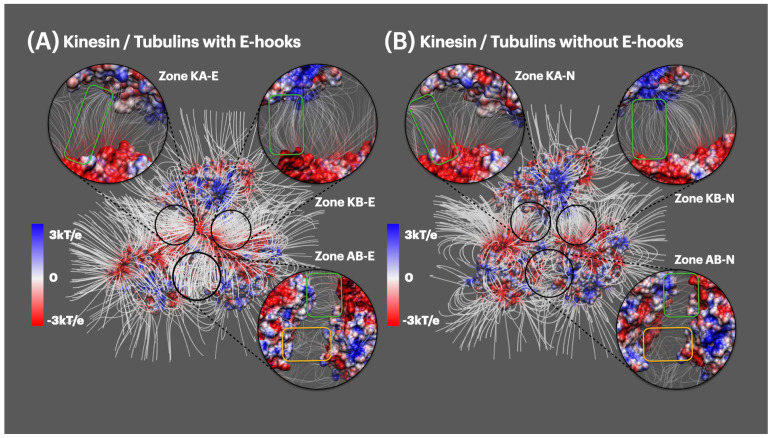
Electric field lines representations. (**A**) Kinesin and tubulins with E-hooks, with the closeup views of interfacial areas of kinesin and alpha tubulins (Zone KA-E), kinesin and beta tubulin (Zone KB-E), alpha and beta tubulin (Zone AB-E); (**B**) Kinesin and tubulins without E-hooks, with the closeup views of interfacial areas of kinesin and alpha tubulin (Zone KA-N), kinesin and beta tubulin (Zone KB-N), alpha and beta tubulin (Zone AB-N). Color scale: −3.0 to 3.0 kT/e (red to blue).

By comparing Figure 5A,B, the overall field line distributions of both complexes are very similar: field lines densities within the interfacial regions (six zones in Figure 5 black circles) are higher than the rest of the areas. While there are remarkable differences noticed when comparing the closeup views of interfacial areas (black circles in Figure 5).

First of all, when comparing the interfacial area between kinesin and alpha tubulin (zone KA-E in Figure 5A and KA-N in Figure 5B), we noticed that the E-hook in zone KA-E makes its surrounding field lines connected to kinesin (green circle in zone KA-E), while in zone KA-N alpha tubulin has a bunch of field lines disconnected to kinesin that are diverged to the outside (green circle in zone KA-N). Secondly, when comparing the interfacial area between kinesin and beta tubulin (zone KB-E in Figure 5A and KB-N in Figure 5B), we noticed that the E-hook makes the field lines density higher especially in green circle regions. Thirdly, when comparing the interfacial area between alpha tubulin and beta tubulin (zone AB-E in Figure 5A and zone AB-N in Figure 5B), it was observed that E-hook makes the surrounding field lines denser (green circles in zone AB-E and zone AB-N). Moreover, except for the local surrounding areas of E-hooks, the slightly further away areas (yellow circles in zone AB-E and zone AB-N) tend to form denser field lines under the influence of E-hook. This fact shows that E-hooks not only influence their own local regions but also affect the further surrounding areas to be more active in binding interactions.

#### 2.4.2. Electric Field Lines between Kinesin and Beta Tubulin

**Figure 6:**
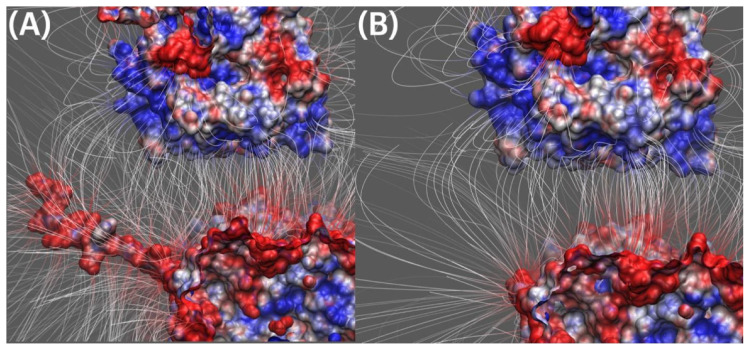
Field lines between kinesin and beta tubulin. (**A**) Kinesin and beta tubulin with E-hook; (**B**) Kinesin and beta tubulin without E-hook. Color scale: −3.0 to 3.0 kT/e (red to blue).

Previously, we discussed the overall field line differences between kinesin and tubulins above in Section 2.4.1. Here, in Figure 6, it is a close-up side view of the interfacial area between kinesin and beta tubulins. Figure 6 shows remarkable field line differences in two complexes: we firstly found the field lines density difference in the E-hook local areas, and also in the surrounding interfacial areas (not the E-hook local areas). This fact shows that E-hooks are not only able to provide their own negative charges but also involved in affecting their surrounding areas to be more negatively charged. Since kinesin is overall positively charged, this mechanism helps to make tubulins more attractive to kinesins.

### 2.5. Electrostatic Forces in the Binding Process

**Figure 7:**
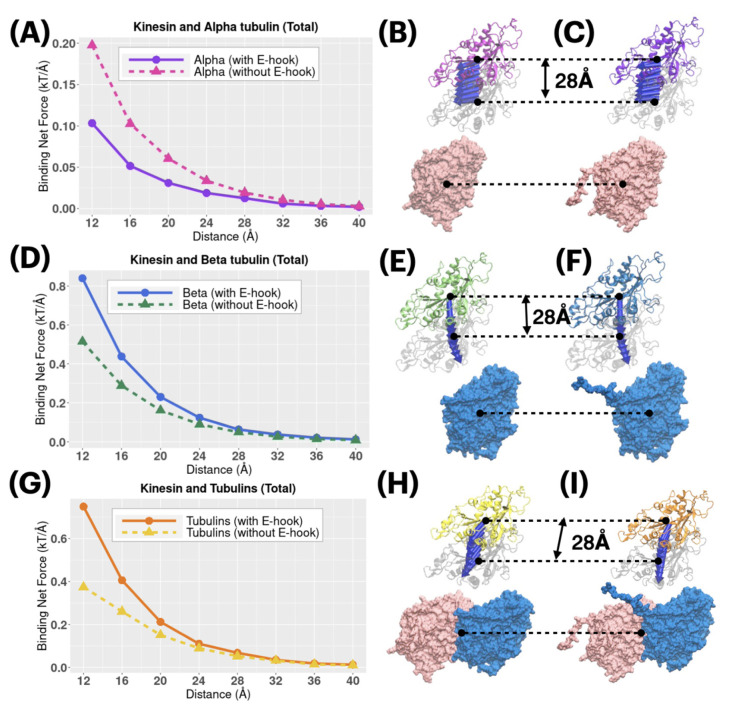
The electrostatic binding forces between kinesin and tubulins at different distances from 12 to 40 Å with the step size of 4 Å. (**A**) Binding net force strengths between Kinesin and Alpha tubulin (with/without E-hooks); (**B**) Binding net force directions between Kinesin and Alpha tubulin without E-hook; (**C**) Binding net force directions between Kinesin and Alpha tubulin with E-hook; (**D**) Binding net force strengths between Kinesin and Beta tubulin (with/without E-hooks); (**E**) Binding net force directions between Kinesin and Beta tubulin without E-hook; (**F**) Binding net force directions between Kinesin and Beta tubulin with E-hook; (**G**) Binding net force between kinesin and tubulin dimer (with/without E-hooks); (**H**) Binding net force directions between kinesin and tubulin dimer without E-hook; (**I**) Binding net force directions between kinesin and tubulin dimer with E-hooks. The blue arrows in Figure 7B,C,E,F,H,I represent the force directions and they were normalized to be of the same size for a better visualization.

Electrostatic forces between kinesin and tubulins were calculated by DelPhiForce (Figure 7). Blue arrows in Figure 7 are shown to visualize the net forces between kinesin and a tubulin (alpha or beta) by shifting the tubulin away from kinesin by variable distances from 12 to 40 Å with the step size of 4 Å. The direction of arrows represents the force direction. Note here, in order to better visualize the directions of the net forces, the magnitudes of the net forces were normalized to be of the same size at different distances, which means that the size of the arrows does not represent the force strength. The force magnitudes are illustrated in Figure 7A,D,G.

From Figure 7B,C,E,F, alpha tubulin mainly provides sliding forces to kinesin while beta tubulin mainly provides attractive forces to kinesin. From Figure 7H,I, the tubulin dimer provides attractive forces to kinesin. Those two facts show that tubulin dimer provides strong attractive forces to kinesin, while alpha and beta tubulin play different roles in providing the electrostatic forces in the binding process. Please note that Figure 6 describes the total forces between Kinesin and Tubulins, and the X-Y-Z components of the total forces are illustrated in Figure S1.

From Figure 7A and Figure S1D,G,J, Alpha without E-hooks have about twice the force strength than Alpha with E-hooks at local distance (12–16 Å), in the total calculation and all components of XYZ directions, while at the longer distance (20–40 Å) the differences between Alpha with or without E-hooks are less and less significant with the distance increasing. This fact shows that E-hooks decrease the distance-sensitivity of binding forces between Alpha tubulin and kinesin. Furthermore, as discussed in the force directions previously, Alpha mainly provides the sliding forces to the kinesin, which helps to adjust the position of kinesin. Here, we demonstrated the E-hook on Alpha tubulin functions as a regulator to limit the strength of sliding forces, which helps the kinesin to be more accurately bound to the binding site on tubulin dimer.

From Figure 7D and Figure S1E,H,K, Beta with E-hooks have stronger force strengths than Beta without E-hooks at both local distances and long distances, especially in Z direction. Since Beta tubulin mainly provides the attractive binding forces between tubulin and kinesin, this fact shows that Beta E-hook helps increase the binding force in the whole binding process. By comparing Figure 7A,D, Alpha and Beta are proven again to play different roles in kinesin–tubulin binding process by helping in different aspects.

## 3. Methods

In this work, we applied multi-scale computational methods, including tools and algorithms in our analyses to study the E-hooks and the binding processes to kinesins. Several software packages were implemented: DelphiForce [27] for electrostatic force calculations, Delphi [28] for electrostatic potential calculations, NAMD2 [29] for MD simulations of E-hook flexibility analyses, etc. Furthermore, we used Euclidean distance in the E-hook flexibility calculations.

### 3.1. Structure Preparation

The original complex structures of kinesin/tubulins and tubulins (with E-hooks sequences) were downloaded from the Protein Data Bank (PDB ID: 6TA3 [30] and 3J71 [31]). Since 6TA3 does not include E-hooks sequences and 3J71 does not include a kinesin structure, we matched those two structures and visualized using Chimera [32]. E-hook structures from alpha and beta tubulin are not available in any of the experimental results, so we applied the MODELLER tool [33] to predict the structure of missing parts (alpha tubulin: GVDSVEGEGEEEGEEY (16 AAs), beta tubulin: DEQGEFEEEEGEDEA (15 AAs)). After those preparations, we got a full complex structure of kinesin and tubulins with E-hooks (Figure 1A).

For the convivence and accuracy of the following calculations, we deleted all the H_2_O (water) molecules that are involved in the original structure. Instead, the implicit solvent model (Poisson–Boltzmann) was utilized in our methods like DelPhi and DelPhiForce.

### 3.2. Molecular Dynamic (MD) Simulations

To observe the motilities and other features of those models, we carried out Molecular Dynamic (MD) Simulations based on six different complex structures (Movie 1–6, generated by Visual Molecular Dynamics(VMD) [34]).For the software, we used NAMD2 [29]; for the hardware, we involved GPUs on Lonestar5 (accessed on 6 December 2021) and CPUs on Stampede2 (accessed on 10 December 2021) clusters at Texas Advanced Computing Center (TACC https://www.tacc.utexas.edu/ (accessed on 6 December 2021)).

A 20,000-step minimization was performed for each simulation, followed by 20 million steps, during which 4000 frames were saved from six 20 ns simulations separately, that is, 1.0 fs per step, 5000 steps per 1 frame, 20 million steps in total. During the MD simulations, the temperature was set to be 310 K, and the pressure was set to be standard using the Langevin dynamics.

### 3.3. Flexibility and Correlation of Alpha and Beta E-Hooks

For the analysis of E-hooks flexibility, we used the Euclidean distance between points, which in Euclidean space is the length of the line segment between the points. We calculated from the Cartesian coordinates of the points using the Pythagorean theorem, with the following procedures:

Let

$$\left(x_{i},\;y_{i},\;z_{i}\right):i=1,..,n$$

be a collection of points. So, the centroid is

$$\left(x,\;y,\;z\right)=\frac{\sum_{i=1}^{n}\left(x_{i},\;y_{i},\;z_{i}\right)}{n}$$

Then, we take

(1) $${\;\sigma }^{2}=\frac{\sum_{i=1}^{n}\left({\left(x-x_{i}\right)}^{2}+{\left(y-y_{i}\right)}^{2}+{\left(z-z_{i}\right)}^{2}\right)}{n}$$

where σ is the standard deviation in Euclidean distance of all points from the centroid, which we define as the flexibility of E-hooks. The results are shown in Figure 2. 

We also calculated the correlation between the movements of Alpha and Beta E-hooks. We selected the C-alpha atoms of the residue at the end of E-hooks (Y451 in Alpha tubulin and A445 in Beta tubulin) at each frame of our MD simulations (41 frames in total, frame No. 0–40). We set the C-alpha location at frame 0 as the reference location, and calculated the distance between the locations of current frame and the location of frame 0. Then, we visualized the distances and the correlation line between two C-alpha atoms in Figure S2, which shows that Alpha E-hook and Beta E-hook have a strong movement correlation with an R-value of 0.7012 under 95% confidence interval level.

### 3.4. Electrostatic Potential Calculations

To study electrostatic features of kinesin and tubulins, we utilized DelPhi [28,35] to calculate the electrostatic potential. In the framework of continuum electrostatics, DelPhi calculates the electrostatic potential ϕ (in systems comprised of biological macromolecules and water in the presence of mobile ions) by solving the Poisson–Boltzmann equation (PBE):

(2) $$\nabla \cdot \left[\epsilon \left(r\right)\nabla \phi \left(r\right)\right]=-4\pi \rho \left(r\right)+\epsilon \left(r\right)\kappa^{2}\left(r\right)\sinh\left(\phi \left(r\right)/k_{B}T\right)$$

where ϕ(r) is the electrostatic potential, є(r) is the dielectric distribution, ρ(r) is the charge density based on the atomic structures, κ is the Debye–Huckel parameter, k_B_ is the Boltzmann constant, and Τ is the temperature. Due to the irregular shape of macromolecules, DelPhi uses a finite difference (FD) method to solve the PBE.

Before the DelPhi calculations, the PQR file of kinesin and tubulins were generated by PDB2PQR [36] tool. We used AMBER force field for PDB2PQR calculation, and removed water molecules. For better results, we ensured the new atoms are not rebuilt too close to existing atoms and optimized the hydrogen bonding network by using a default PDB2PQR setting.

For the calculation parameters in DelPhi, the grid resolution was set to be 1.0 grids/Å. The dielectric constants were set as 2.0 for protein and 80.0 for the water environment, respectively. The probe radius for generating the molecular surface was 1.4 Å. Salt concentration was 0.15 M. The boundary condition for the Poisson Boltzmann equation was set as a dipolar boundary condition. After the calculation, the values of electrostatic potential on the surface were visualized with Chimera (Figure 3). In order to visualize electric field lines between kinesin and tubulins and to get the best visualization, we separated the alpha tubulin and the beta tubulin by 20 Å using StructureMan [37], and the separated kinesin from them(the separated tubulins) by 20 Å. VMD was implemented based on the electrostatic potential map from DelPhi calculations and the color scale range was set from −3.0 to 3.0 kT/e. The results of electrostatic potetial calculation are shown in Figure 3 and Figure 4, and the visuialization of electric field lines are shown in Figure 5 and Figure 6. 

### 3.5. Electrostatic Binding Force Calculations

To compare the strengths and directions of electrostatic forces between kinesin and tubulins, DelPhiForce [28,29] was implemented to perform the force calculations. In order to study the binding process from a long distance (40 Å) to a local distance(12 Å), we separated the kinesin from tubulin dimer in the direction of their mass centers connection line with the distances ranging from 12 Å to 40 Å with a step size of 4 Å in Figure 7. For the calculation in alpha-Kinesin complexes (with/without E-hooks), we removed the residues of the beta tubulin; for the calculation in beta-Kinesin complexes (with/without E-hooks), we removed the residues of the alpha tubulin. The electrostatic binding forces calculated by DelphiForce were visualized with VMD and represented by same-size arrows.

## 4. Limitation

One limitation of our analyses is the model size. We used the most basic model (one single tubulin dimer) and E-hook of alpha tubulin in this model is more flexible than E-hook of beta due to the location restriction. However, for the basic analyses within a local area of a kinesin’s one single walking step, our model is appropriate and reliable. Some further analyses, based on a larger system (nine tubulin dimers) to explain the kinesin walking mechanisms (beyond one single step), will be studied in our future research.

## 5. Conclusions

As an important type of microtubules-based motors, kinesin plays an essential role in cargo transport within cells. The C-terminal tails (E-hooks) of alpha and beta tubulins of microtubules have been proven to be helpful to the binding between kinesin and tubulins [25].

In our work, we implemented multi-scale computational methods in E-hook-related analyses, including flexibilities of E-hooks, binding force calculations at binding interfaces between kinesin and tubulins, and electrostatic potential calculations on the surface of kinesin and tubulins. Our results show that E-hooks have several functions during the binding process: E-hooks utilize their own high flexibility in a solvent to increase the chances of finding a kinesin, based on the E-hook flexibility analyses; E-hooks help tubulins to be more attractive to kinesin, based on the electrostatic calculations. Besides, we also observed the differences between alpha and beta tubulins: beta tubulin shows higher flexibility than alpha tubulin, based on the E-hooks flexibility analyses; beta tubulin generates higher attractive forces (about twice the strength) to kinesin at different distances, no matter with E-hooks in the structure or not, based on the force calculations. Those facts may indicate that compared to alpha tubulin, beta tubulin contributes more to catching and attracting a kinesin to the microtubule.

## Data Availability

Not applicable.

## Supplementary Materials

The following supporting information can be downloaded at: https://www.mdpi.com/article/10.3390/ijms23042035/s1.
