# Supplementary material for: Computational Study on E-Hooks of Tubulins in the Binding Process with Kinesin
Source: Int J Mol Sci. 2022 Feb 12;23(4):2035. doi: 10.3390/ijms23042035 (PMC8877516; doi:10.3390/ijms23042035)
Supplement: Supplementary file 1 [file ijms-23-02035-s001.zip › ijms-1561732-supplementary/Supplementary.pdf]

Computational Study on E-hooks of Tubulins in the Binding Process with Kinesin

Yixin Xie[1], Lin Li[1,2*]

[1] Computational Science Program, The University of Texas at El Paso, El Paso, TX

[2] Department of Physics, The University of Texas at El Paso, El Paso, TX

***Correspondence:**

Lin Li: lli5@utep.edu

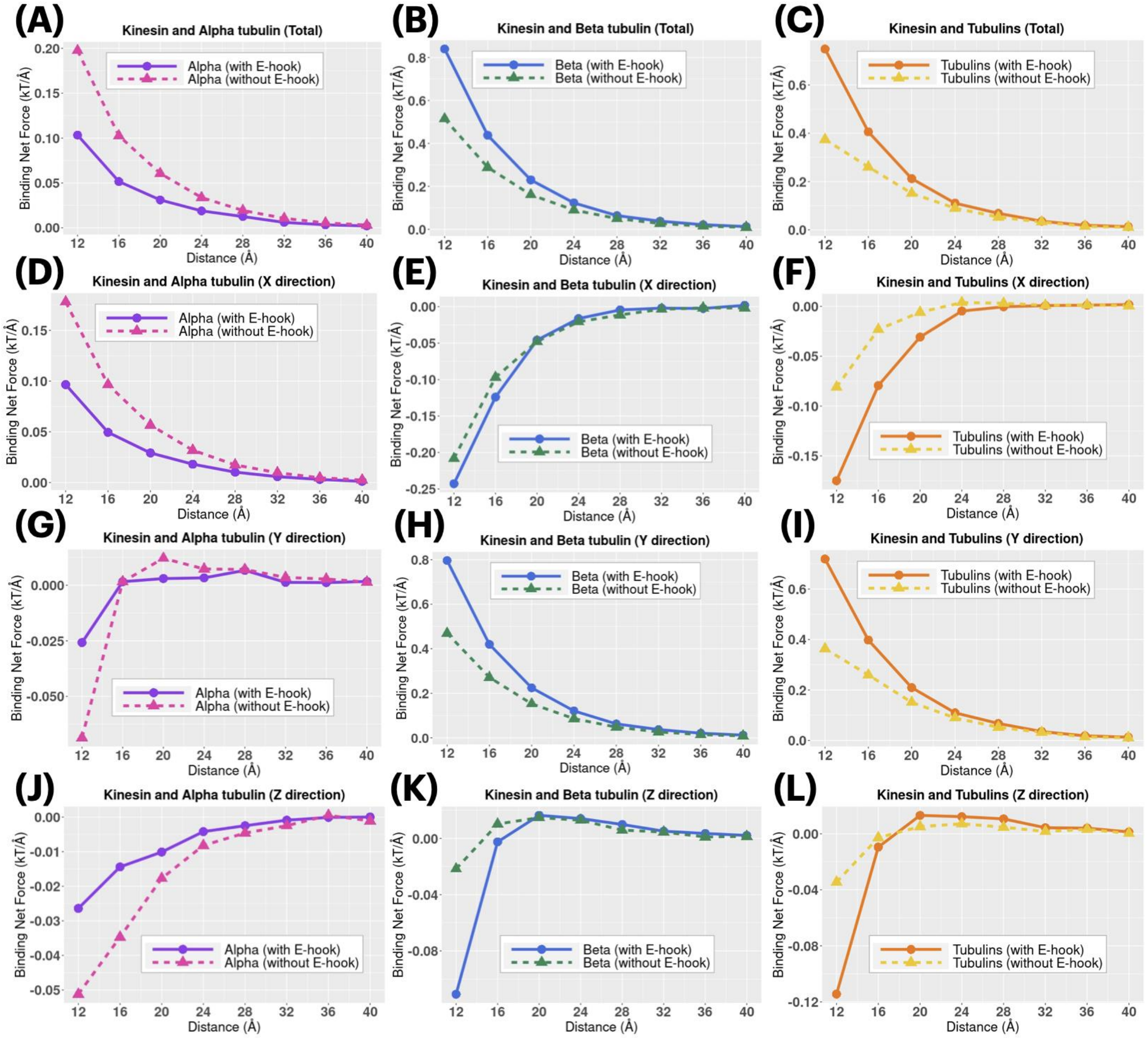

Figure S1. Electrostatic Binding Forces between Kinesin and Tubulins. (A) Total electrostatic binding forces between Kinesin and Alpha Tubulin; (B) Total electrostatic binding forces between Kinesin and Beta Tubulin; (C) Total electrostatic binding forces between Kinesin and Tubulin dimer; (D) Electrostatic binding forces between Kinesin and Alpha Tubulin in X direction; (E) Electrostatic binding forces between Kinesin and Beta Tubulin in X direction; (F) Electrostatic binding forces between Kinesin and Tubulin dimer in X direction; (G) Electrostatic binding forces between Kinesin and Alpha Tubulin in Y direction; (H) Electrostatic binding forces between Kinesin and Beta Tubulin in Y direction; (I) Electrostatic binding forces between Kinesin and Tubulin dimer in Y direction; (J) Electrostatic binding forces between Kinesin and Alpha Tubulin in Z direction; (K) Electrostatic binding forces between Kinesin and Beta Tubulin in Z direction; (L) Electrostatic binding forces between Kinesin and Tubulin dimer in Z direction. As mentioned in method section 2.5, for the calculation in Alpha-Kinesin complexes (with/without E-hooks), we removed the residues of Beta tubulin; for the calculation in Beta-Kinesin complexes (with/without E-hooks), we removed the residues of Alpha tubulin.

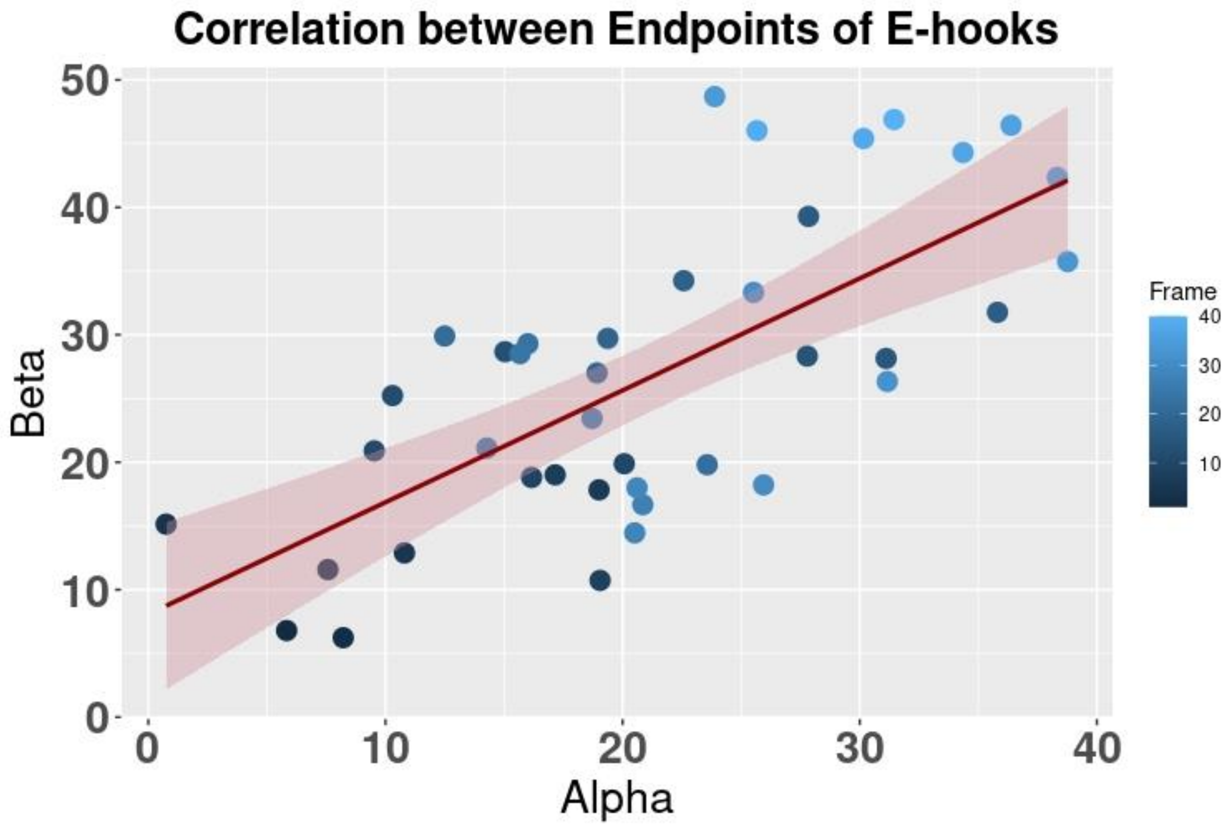

Figure S2. Movement correlation between E-hooks of Alpha and Beta tubulins. We set the location of each tubulin endpoint at frame 0 (the initial location) as a reference and calculated the distance between the location of frame 0 and the current. Red line is the correlation line and dots are the distance values at frame 0 to 40. Pink area is the confidence interval of the correlation line with the interval level of 95%. R value is 0.7021.
